# Comparison between an Emerging Point-of-Care Tool for TSH Evaluation and a Centralized Laboratory-Based Method in a Cohort of Patients from Southern Italy

**DOI:** 10.3390/diagnostics11091590

**Published:** 2021-08-31

**Authors:** Alfredo Di Cerbo, Nazario Quagliano, Antonella Napolitano, Federica Pezzuto, Tommaso Iannitti, Alessandro Di Cerbo

**Affiliations:** 1 Leonardo da Vinci Private Clinic, Via Nicola De Dominicis, 71121 Foggia, Italy; alfredo.dicerbo@gmail.com (A.D.C.); f.pezzuto@hotmail.it (F.P.); 2 Biometron Laboratory, Viale Aldo Moro, 122/128, 71011 Apricena, Italy; nazario.quagliano@virgilio.it (N.Q.); biometron.lab@virgilio.it (A.N.); 3 Independent Researcher, Southampton SO16 0BS, UK; 4 School of Biosciences and Veterinary Medicine, University of Camerino, Via Circonvallazione 93/95, 62024 Matelica, Italy

**Keywords:** endocrine and metabolic disorders, thyroid stimulating hormone, point-of-care test

## Abstract

Endocrine and metabolic disorders are a common condition in Europe and worldwide, and, among these, thyroid dysfunction still remains a problem. The measurement of thyroid stimulating hormone (TSH) levels represents the first-line assay for the assessment of thyroid function. In the present study, we compared serum concentrations of TSH, measured using a commercially available point-of-care test (POCT) method (FastPack^®^ IP) and an established “conventional” laboratory-based method (Beckmann Access 2) in a cohort of patients from Foggia in Southern Italy. A strong correlation (*r* = 0.994) was found between both methods and was also confirmed by receiver operating characteristic (ROC) curve analysis (0.82). The within-run coefficient of variation (CV) using FastPack^®^ ranged from 4.03% and 8.57% at the TSH concentrations of 39.49 and 0.70 mIU/L, respectively. The between-run CV was 10.34% and 6.33% at the TSH concentrations of 0.87 and 26.55 mIU/L, respectively. The ratios of within- to between-assay CV were 0.83 and 1.06 at the TSH levels of 0.70 and 52.59 mIU/mL, respectively. In this study, we showed that serum TSH levels can be measured in a few minutes and at low-cost in terms of materials and equipment required. We observed that this approach is user-friendly, accurate, reproducible, and suitable for use in the clinic, while also meeting the criteria for effectiveness, impact, efficiency, and sustainability.

## 1. Introduction

Endocrine and metabolic disorders are a common condition in Europe and worldwide [1,2,3]. They result in a costly use of health resources and a loss of productivity [4,5,6,7]. The prevalence and incidence of certain diseases such as thyroid disorders [8,9], diabetes [3], obesity [10], hyperparathyroidism [11], osteoporosis [12], and vitamin D deficiency [13] have been defined in several screening programs and population-based studies. Iodine deficiency, defined as a daily iodine intake inferior to 50–100 μg, is the most common cause of thyroid disorders and triggers the growth of the thyroid gland, leading to goiter and thyroid nodule formation and, if untreated, hypothyroidism [14]. The prevalence of goiter in iodine-deficient areas can be as high as 70–90% [15,16,17,18]. Iodine is essential for brain development during fetal life and the neonatal period [19]. The most serious complication of congenital iodine deficiency is endemic cretinism, characterized by severe mental retardation, neurological symptoms, and/or hypothyroidism [19]. In addition, iodine deficiency negatively affects quality of life and economic productivity [20]. 

In the last decades, iodine prophylaxis programs, which are based on salt or water iodization and implemented by the World Health Organization and in Italy (Law No. 55/2005), have not only decreased the prevalence of iodine deficiency disorders but have also caused major changes in the steady pattern of thyroid disorders; these are characterized by an increased prevalence of autoimmune thyroid disease, ranging from atrophic to goitrous Hashimoto’s thyroiditis, Graves’ hyperthyroidism, non-autoimmune hyperthyroidism, and autonomous thyroid nodules [9,20,21,22]. Thus, thyroid dysfunction still remains a problem. Moreover, high resolution ultrasound allows the assessment of thyroid size and morphology as well as the detection of small and non-palpable thyroid nodules, allowing subjects suffering from thyroid problems to be diagnosed by primary care physicians.

Point-of-Care Tests (POCTs) are generally utilized outside the laboratories to directly evaluate a number of clinical parameters. These diagnostic tools affect the quality and rapidity of care and allow the design of a patient-centered care approach [23,24]. 

Unlike other laboratory tests, whose results can take several hours to a few days to become available, POCTs reduce analysis time to a few seconds or minutes, being helpful especially in emergency conditions. 

In the last 15 years, some POCTs for thyroid stimulating hormone (TSH) measurement have been developed and proposed [25,26,27,28,29], suggesting the need to compare these tools to standard laboratory approaches. In the present study, we aimed to firstly compare serum concentrations of TSH, measured by means of a commercially available POCT method (FastPack^®^ IP System, Qualigen Therapeutics, Inc., Carlsbad, CA, USA) and an established “conventional” laboratory-based method in a cohort of patients and, secondly, to assess the convenience of carrying out the POCT analysis instead of the laboratory-based one, in terms of patience compliance.

## 2. Materials and Methods

### 2.1. Study Design

All subjects provided a written informed consent and completed a questionnaire concerning their compliance with the point-of-care test POCT FastPack^®^ IP System and the Access 2 Beckman Coulter method to participate in this interventional, single-center, open-label study. Inclusion criteria were age > 18 years and stable disease or healthy status. Exclusion criteria were any acute or chronic condition that would limit the ability to participate in the study, pregnancy, breastfeeding state, or refusal to provide written informed consent. The study was conducted in accordance with the ethical principles for medical research involving human subjects, as set forth in the World Medical Association Helsinki Declaration of October 2013. The study was approved by the local Ethics Committee at Foggia (Italy) (Reference Number 26/SegCE/2020 of 27 April 2020). Once accepted to participate in the study, each patient underwent a single blood withdrawal at the central laboratory (Biometron, Apricena, Italy) to assess TSH values by means of FastPack^®^ IP System compared to the ACCESS2 Beckman Coulter instrument. Unless otherwise indicated, 100 microliters of serum were utilized in both the FastPack^®^ IP System and the Access 2 Beckman Coulter assay. The present study includes the following endpoints, biochemical measurements, and quality control.

### 2.2. Biochemical Measurements

#### 2.2.1. FastPack^®^ IP TSH Immunoassay

The FastPack^®^ IP TSH immunoassay is quantitative chemiluminescence assay based on the “sandwich” principle. Briefly, a mixture of a biotinylated monoclonal TSH-specific antibody and a monoclonal anti-TSH antibody labeled with alkaline phosphatase reacts with TSH from a 100 μL sample. During a secondary incubation, streptavidin-coated paramagnetic particles react with the TSH-antibody complex via the interaction between biotin and streptavidin. After washing off the unbound material, a chemiluminogenic substrate is added and light is measured. The intensity of light produced is directly proportional to the TSH concentration in the sample.

#### 2.2.2. Beckman Access 2 TSH Immunoassay

The Access 2 TSH immunoassay is an immunoenzymatic sandwich assay. Briefly, a mixture of a mouse anti-human TSH antibody labeled with alkaline phosphatase and a mouse anti-human TSH monoclonal antibody adsorbed on paramagnetic particles reacts with two different sites of TSH from a 100 μL sample. Following incubation, the chemiluminogenic substrate is added and light is measured. The intensity of light produced is directly proportional to TSH concentration in the sample.

### 2.3. Instrument Description

**Figure 1:**
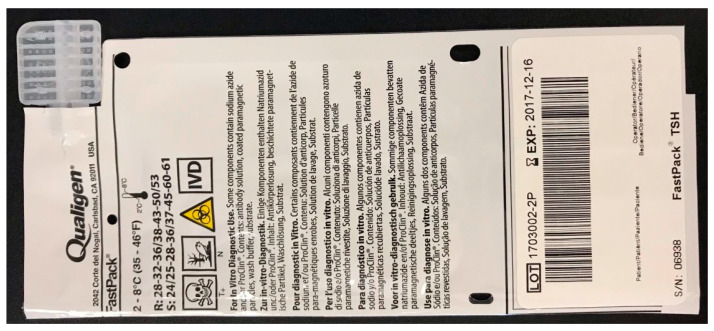
Example photograph of a TSH pack displaying the bar code.

The FastPack^®^ IP System is a rapid immunoassay testing system including the FastPack^®^ IP immunoassay analyzer and the FastPack^®^ IP test pouch [30]. The FastPack^®^ IP System performs a series of complex software-controlled operations ranging from reading the bar code attached to the individual test pouch to the measurement of light produced in the final reaction (Figure 1).

### 2.4. Quality Control

In order to evaluate the reproducibility and stability of the FastPack^®^ IP System assay, we preliminarily implemented a quality control process. The quality control protocol, determined according to the NCCLS EP6-A guideline [31,32], was based on (i) assessment of the within- and between-assay variation; (ii) parallelism on the dilution test; and (iii) the recovery test. For the assessment of the within-run precision, 10 replicates of sera coming from 2 patients with high TSH, 1 patient with intermediate TSH, and a pool of sera from 3 patients with low TSH levels were sequentially run each in the same series and within the same day. 

The between-run repeatability was evaluated calculating the coefficient of variation of 14 replicates of two control samples, which were run on separate days. We also calculated the ratio of within- to between-assay CV, which is considered an index of the temporal stability of the system [32,33].

Accuracy was checked with dilution/parallelism and recovery tests at the same time that precision was being evaluated. To perform a dilution study, patient’s serum from a hypothyroid patient was serially diluted with TSH-free (TSH 0.001 mIU/L) serum from a patient with active Graves’ disease. Dilutions, covering an interval from 36.87 to 0.97 mIU/mL, were selected so that they were falling in the range of TSH levels, which guarantees a low statistical error as previously indicated by the within-run precision test.

All samples were run in triplicate and in parallel on the FastPack^®^ IP and on the Beckman Access 2 systems, respectively. The observed values uncorrected for dilution were plotted against the dilution or, alternatively, the final concentrations were multiplied by the appropriate dilution factor and the results were plotted against dilution.

The recovery test is most often used in the clinical chemical literature. The objective of a recovery study is to test whether a known increment of analyte added to a sample can be measured quantitatively by the assay being examined. For the recovery test we previously analyzed the TSH level both in base and spike samples alone, and then we measured it in triplicate in a single-run patients’ serum opportunely mixed to obtain base plus spike TSH levels covering a low, intermediate, and high TSH concentration range. Samples to be measured were obtained by adding fifty microliters of spike sample to fifty microliters of base sample to obtain TSH concentrations ranging from 1.09 to 41.2 mIU/L. Percent recovery (R%) was calculated according to the formula: R% = (amount observed/amount expected) × 100, where amount expected = amount in base + amount added.

### 2.5. Study Population

Sera to be tested for TSH were obtained from 72 patients and 28 control subjects (32 males, 68 females, aged 19–90 years) coming to Biometron Laboratory (Apricena, Foggia, Italy). The serum was separated from the cells by centrifugation (3000 RPM for 10 min), and the serum was used for Beckman Access 2 and FastPack^®^ IP analyses.

All 100 subjects participating in the study underwent a thorough medical examination and thyroid ultrasound. All subjects were asked about their personal and family history of thyroid and autoimmune disorders, eating and smoking habits, any past or current therapy with special attention paid to L-T4 and any other drug that could affect thyroid function, recent exposure to iodinated contrast media, and previous radiation treatment to the head, neck, or chest.

Thyroid ultrasound was performed by a single operator using the SonoSite MicroMaxx portable ultrasound system equipped with HFL38 [6–13 MHz] linear transducer (SonoSite, Inc., Bothell, WA, USA). Thyroid volume was calculated according to the formula of the ellipsoid model (length × width × thickness multiplied by the correction factor π/6) for each lobe [34].

### 2.6. Questionnaire Data

All subjects were asked to complete an anonymous questionnaire concerning their compliance with receiving the medical examination immediately after biochemical analyses results (<15 min) achieved by means of FastPack^®^ IP with respect to the 2-day response time required by Beckman Access 2.

### 2.7. Statistical Analysis

Data were analyzed using GraphPad Prism 8 software (GraphPad Software, Inc., La Jolla, CA, USA). To evaluate the normal distribution of patients’ age, TSH levels, and thyroid volumes, normality tests were preliminarily performed using the Anderson–Darling test, the D’Agostino & Pearson test, the Shapiro–Wilk test, and the Kolmogorov–Smirnov test. Continuous variables are presented as mean ± SD or median (25/75 percentile) and compared using parametric or non-parametric tests, according to normality test results. Categorical variables are expressed as percentages, and the differences were analyzed using the chi-square (Fisher’s exact) test. The coefficient of variation (CV%) and relative standard deviation (RSD%) were calculated according to the formula (SD/mean) × 100, while accuracy was evaluated by means of the coefficient of correlation (coefficient of determination (*r*^2^) and Bland–Altman methods). We also compared FastPack^®^ IP and Beckman Access 2 methods in terms of sensitivity and specificity by means of receiver operating characteristic (ROC) curve and/or area under curve (AUC). * *p* < 0.05 was considered significant.

## 3. Results

### 3.1. Quality Control

The results of the within- and between-run reproducibility tests are shown in Table 1.

**Figure 2:**
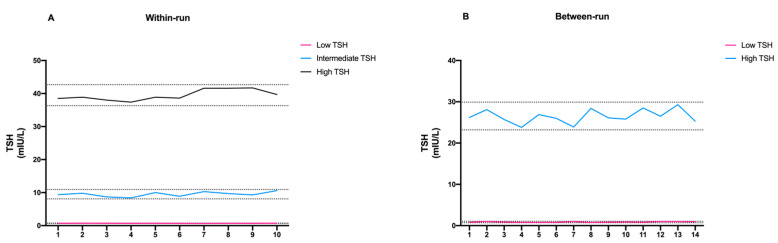
Schematic representation of (**A**) within-run and (**B**) between-run precision of FastPack^®^ IP results.

The within-run CV ranged from 4.03% and 8.57% at the TSH concentrations of 39.49 and 0.70 mIU/L, respectively. The between-run CV was 10.34% and 6.33% at TSH concentrations of 0.87 and 26.55 mIU/L, respectively. The ratios of within- to between-assay CV were 0.83 and 1.06 at TSH levels of 0.70 and 52.59 mIU/mL, respectively. The experimental results obtained from both within- and between-run assays were then evaluated for the presence of possible outlier data. To do so, we decided to set the tolerance limit at ±2 SD (95% confidence limit) and plot the individual results to see if there were any values outside the set limits. As shown in Figure 2, all data fell within the tolerance limit.

### 3.2. Dilution/Parallelism Assay

**Figure 3:**
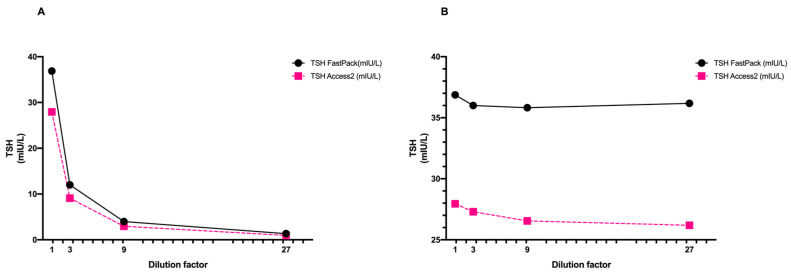
Schematic representation of high-TSH patient’s sample dilutions expressed as (**A**) raw data and (**B**) after multiplying by dilution factor. TSH levels were measured using FastPack^®^ system and Access 2 Beckman reference method.

The serum used for the dilution assay was serially diluted with a TSH-free serum starting from a TSH level of 36.87 mUI/L to a final concentration of 1.07 mUI/L. When the observed results were plotted against the dilution, or when TSH levels were multiplied by the dilution factor, we observed a similar trend between the patient’s sample and the reference sample (Figure 3A,B).

### 3.3. Recovery Test

Percent recovery, as shown in Table 2, ranged from 89.0 to 102.0% at TSH concentrations of 1.09 and 39.4 mIU/L, respectively. Table 1 also shows that all data were within 80 and 120%, which is considered the acceptance range for the recovery test [35].

### 3.4. Comparison between FastPack^®^ IP and Access 2 TSH Immunoassays

**Figure 4:**
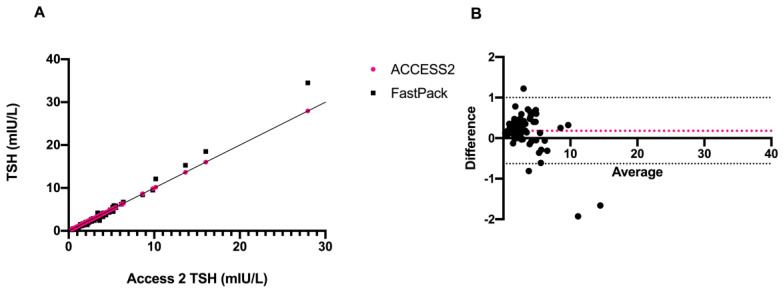
Schematic representation of (**A**) correlation and (**B**) Bland–Altman plot of TSH levels of Access 2 vs. FastPack^®^ IP.

The initial aim of the study was to compare the FastPack^®^ IP TSH immunoassay with the established conventional laboratory-based Access 2 Beckman method. Thus, after the encouraging results of preliminary quality control studies, we compared TSH levels obtained with the two different laboratory methods. A strong correlation (*r* = 0.994) was found between the two sets of data (Figure 4A,B).

**Figure 5:**
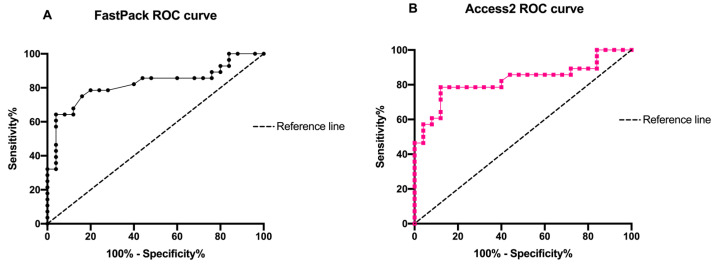
Schematic representation of (**A**) FastPack^®^ IP and (**B**) Access2 ROC curve of TSH concentrations.

At the same time, such correlation was also confirmed by the ROC curves (0.82 for FastPack^®^ IP and Access 2) as shown in Figure 5.

### 3.5. Study Population

A total of 100 subjects agreed to participate in the study and were enrolled from November 2020 to April 2021. The 100 subjects recruited for the study were divided into groups based on physiological status or thyroid disease (Table 3).

Twenty-five did not have any disease and were used as controls. Forty-five patients were suffering from Hashimoto’s thyroiditis (28 untreated, 17 on L-thyroxine replacement therapy), 6 from Graves’ disease, 21 from nonautoimmune thyroid disease (including 6 from uninodular and 15 multinodular nontoxic goiter), and 3 from papillary thyroid carcinoma. Patients with overt hypothyroidism were treated with doses of L-T4 adequate to normalize serum thyrotropin concentrations. Among patients with subclinical hypothyroidism, only individuals with TSH levels > 10 mIU/L, thyroid-antibody positive, or antibody-negative with symptoms related to hypothyroidism, were treated with replacement L-thyroxine therapy. All patients with Graves’ disease were on methimazole as they refused radioiodine therapy. Two Graves’ patients reported having previously received a steroid therapy for active thyroid-associated orbitopathy. None was thyrotropin-receptor antibody positive at inclusion. Six and 15 patients were suffering from uninodular and multinodular non-toxic goiter, respectively. Fine needle aspiration (FNA) was advised to three of these patients. In these subjects, clinical findings and the echographic pattern suggested possible absence of malignancy. All three patients with a history of papillary thyroid carcinoma had undergone thyroidectomy, prophylactic central neck lymph node dissection, and radioactive iodine treatment and were currently being treated with replacement doses of L-thyroxine. When TSH levels were analyzed within individual groups of patients, no significant difference was found between the FastPack^®^ IP and Access 2 Beckman TSH immunoassays.

### 3.6. Prevalence of Goiter

**Figure 6:**
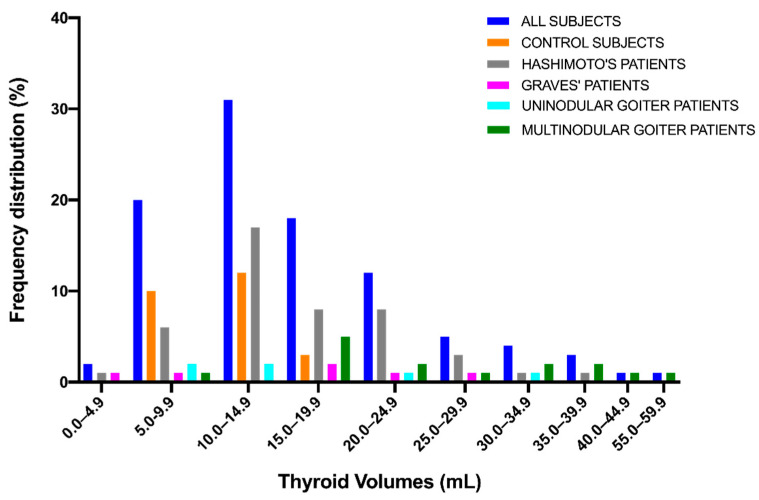
Frequency distribution of thyroid volumes.

Frequency distribution of thyroid volumes for the study population is shown in Figure 6.

Thyroid volumes were not normally distributed (normality tests: Anderson–Darling test, *p* < 0.0001; D’Agostino & Pearson test, **** *p* < 0.0001; Shapiro–Wilk test, **** *p* < 0.0001; Kolmogorov–Smirnov test, **** *p* < 0.0001). The median volume value was 14.0 mL (mean ± SD, 16.6 ± 9.1) (range 2.4–56.0) (Table 3).

**Figure 7:**
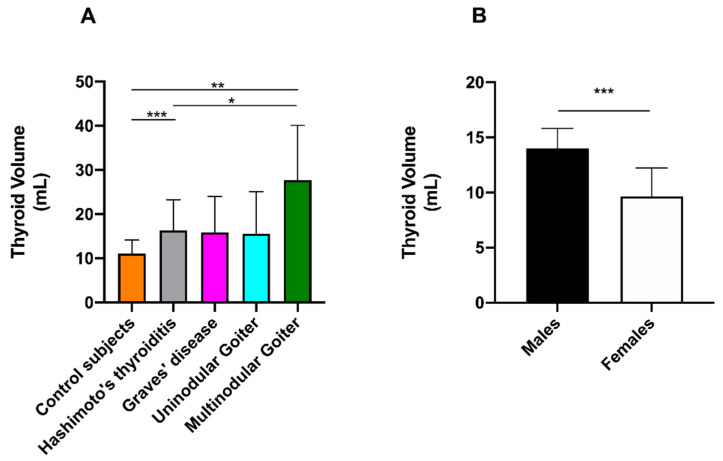
Schematic representation of thyroid volumes in (**A**) different classes of patients and (**B**) between male and female control subjects. * *p* < 0.05, ** *p* < 0.01, and *** *p* < 0.001.

The thyroid volume in control subjects was 11.1 ± 3.1 mL (mean ± SD) and 14.0 ± 1.8 mL in males and 9.7 ± 2.6 mL in females (*** *p* < 0.001). Thus, goiter was diagnosed when thyroid volume was more than 2 SD above the mean thyroid volume of sex-matched control subjects (i.e., greater than 17.6 and 14.9 mL in males and females, respectively). Based on these cut-off values, goiter was diagnosed in 25 out of 66 females (37.9%) and 14 out of 31 males (45.2%). The prevalence of goiter was not significantly different between females and males (Fisher’s exact test, *p* = 0.51) and was not increasing with age (Spearman *r* = 0.1341, *p* = 0.19). A significant difference was found for thyroid volume between control subjects vs. Hashimoto’s patients, control subjects vs. patients with multinodular goiter, Hashimoto’s patients vs. patients with multinodular goiter (Dunnett’s T3 multiple comparison test: *** *p* < 0.001, *** *p* < 0.001, and * *p* < 0.05, respectively) (Figure 7A), and control males vs. females (Mann-Whitney test, *** *p* < 0.001) (Figure 7B) but not between untreated and treated Hashimoto patients (Mann–Whitney test).

### 3.7. Questionnaire Data Results

At the enrolment, most of the patients were used to undertaking TSH analysis three to five times per year, complaining about the long times required to achieve a result when using the laboratory-based instrument. At the same time, all of the patients appreciated the innovative POCT-based analysis approach and, in particular, the shorter time (<15 min) required to achieve the results.

## 4. Discussion

Thyroid gland disorders are common in the general population [36]. Besides iodine deficiency, which is becoming gradually less of a concern due to iodine prophylaxis and food circulation, autoimmune thyroid diseases and thyroid nodules represent the most common thyroid disorders faced by clinical endocrinologists worldwide [1,2,16,17,18,37,38,39]. 

Today, thyroid dysfunction includes diseases ranging from biochemical, asymptomatic subclinical hypo- or hyperthyroidism to overt symptomatic hypo- or hyperthyroidism. Subclinical hypothyroidism, defined as a normal FT4 and moderately elevated TSH levels, affects approximately 5% of women and 3% of men in the United States, and 0.5% of the adult population may be suffering from undiagnosed overt hypo- or hyperthyroidism, characterized by low or high levels of thyroid hormones, respectively [37,38,39,40].

In 2015, the U.S. Preventive Services Task Force (USPSTF) published a study aimed to update the 2004 USPSTF review on the benefits and harms of screening and treatment of subclinical and undiagnosed overt hypothyroidism and hyperthyroidism in adults without goiter or thyroid nodules. The conclusion of the study was that “More research is needed to determine the clinical benefits associated with thyroid screening” [41]. However, it is commonly accepted that the measurement of TSH levels represents the first-line assay for the assessment of thyroid function. Due to the high prevalence of thyroid disorders, rapid diagnosis and treatment are required. Thus, it would be desirable that TSH levels are measured in a rapid way directly at the general practitioners’ and endocrinologists’ offices. This is especially important for patients suffering from subclinical hypo- and hyperthyroidism who may not have symptoms or signs of thyroid dysfunction. However, the TSH assay should be standardized, quality control studies implemented, and repeatability and accuracy ensured.

Since the 1950s, many methodologies have been utilized to measure thyroid hormone as well as TSH levels in plasma or serum. Diagnosis and treatment of thyroid disorders could benefit from improvements in the sensitivity and specificity of in vitro thyroid tests. The serum level of TSH has been evaluated by radioimmunoassay (RIA) since 1965 [42]. The main disadvantage of these earlier assays was low sensitivity (making them unable to detect TSH levels below 0.1 mIU/L), and specificity added to possible cross-reactivity with similar molecules, like LH and FSH, and, especially during pregnancy, hCG. 

Afterwards, other methodologies, including third-generation methods, characterized by a much higher sensitivity (up to ≤0.01–≤0.001 mIU/L), including isotopic (immunoradiometric, IRMA; immunoenzymatic), and non-isotopic methods (like enzyme linked immunosorbent, ELISA; immunofluorometric, FIA; immunochemiluminescence assays, CLIA) have been utilized [43]. Because of its advantages, mainly high sensitivity and specificity as well as a low background signal, automatic and faster sample processing, e.g., CLIA technology, has gradually invaded the immunoassay territory, now being the technology, most frequently utilized to measure hormone levels in serum/plasma. Nevertheless, a significant disadvantage of CLIAs is represented by the high cost of instrumentation.

In this study we utilized a CLIA-based POCT methodology using paramagnetic particles to separate free- from antibody-bound TSH and a small-sized bench-top luminometer to read the intensity of light produced after adding the chemiluminogenic substrate. We showed that serum TSH levels can be measured with precision and accuracy in a few minutes, at low cost in terms of materials and equipment and with good compliance from the patients. Although we acknowledge that biotin plasma levels interfere with SA/B-based systems [44], none of our study subjects showed discrepancies between results of the TSH assay and their clinical presentation. Moreover, none of them reported to be taking dietary supplements or multivitamin formulations containing biotin within the seven days prior to blood withdrawal.

In 2016, Wang et al. published an article describing a new method for qualitative and quantitative measurement of TSH in serum, based on the use of colloidal gold-labeled TSH antibody coated on a microporous membrane [45]. They showed within-assay CVs of 17.84%, 13.92%, and 9.62%, and between-assay CVs of 20.19%, 15.34%, and 8.76%, at TSH concentration of 2.51, 7.63, and 11.08 mIU/L, respectively. While recognizing a precision failure of the method at low TSH levels, they concluded that the immune colloidal method can be acceptable in a simplified screening for hypothyroidism. 

## 5. Conclusions

In the present study we showed that the FastPack^®^ IP assay is a very reliable method for evaluation of low-physiological, intermediate, and high TSH levels. Moreover, from a practical point of view, we observed that it is user-friendly, accurate, reproducible, and suitable for use in the clinic. Finally, in terms of criteria for evaluation, it fulfils the promise of effectiveness, impact, efficiency, and sustainability.

## Figures and Tables

**Table 1:** TSH (mIU/mL) within- and between-run CV and relative standard deviation (RSD) of the FastPack^®^ IP TSH assay. (* Nominal values given by manufacturer.).

Within-Run Assay				
	Low–Normal TSH Level	Intermediate TSH Level	High TSH Level	High TSH Level
Samples	N = 10	N = 10	N = 10	N = 10
Mean ± SD	0.70 ± 0.06	9.51 ± 0.71	39.49 ± 1.59	52.59 ± 3.53
CV (%)	8.57	7.47	4.03	6.71
RSD (%)	0.70 ± 8.57	9.51 ± 7.47	39.49 ± 4.03	52.59 ± 6.71
**Between-Run Assay**				
	**Control Sample 1**		**Control Sample 2**	
Lot#	2001037		2001038	
Mean *	0.89		23.80	
Range *	0.39–1.40		15.8–31.8	
Samples	N = 14		N = 14	
Mean ± SD	0.87 ± 0.09		26.55 ± 1.68	
CV (%)	10.34		6.33	
RSD (%)	0.87 ± 10.84		26.55 ± 6.33	

**Table 2:** Recovery ability of FastPack^®^ IP system at seven different TSH levels.

Sample	TSH Added (mIU/L)	TSH Recovered (mIU/L)	Recovery (%)
#1	1.09	0.97	89.0
#2	3.26	3.07	94.2
#3	9.77	9.67	99.0
#4	21.1	20.4	96.7
#5	29.3	29.8	101.7
#6	39.4	40.2	102.0
#7	41.2	39.6	96.1

**Table 3:** Baseline and clinical characteristics of subjects at the enrollment.

Patients’ Features	Mean ± SD	Median	Range
Age (yr)			
-All subjects (100)	52.2 ± 17.3	51.5	19–90
-Control subjects (25)	47.8 ± 18.3	44	19–79
-Hashimoto’s thyroiditis (45)	51.2 ± 16.7	51	21–86
-Graves’ disease (6)	48.3 ± 11.8	52.5	30–89
-Nodular non-toxic goiter (6)	56.2 ± 20.3	54	28–90
-Multinodular non-toxic goiter (15)	59.1 ± 16.9	52	23–89
-Papillary thyroid carcinoma (3)	69.3 ± 11.9	73	56–79
Sex (F/M)	68/32		
Family history of thyroid disease (Y/N)	41/59		
Smoker (Y/N)	22/78		
Use of iodized salt (Y/N)	61/39		
Radiation treatment to head, neck, or chest (Y/N)	0/100		
Other autoimmune diseases (Y/N)	4/96		
Thyroid volume (mL)			
-All subjects (97)	16.6 ± 9.1	14.0	2.4–56.0
-Control subjects (25)	11.1 ± 3.1	11.4	5.8–16.2
-Hashimoto’s thyroiditis (45)	16.3 ± 7.0	14.1	2.4–35.2
-Graves’ disease (6)	15.9 ± 8.2	18.3	4.3–25.7
-Nodular non-toxic goiter (6)	15.5 ± 9.5	10.7	8.1–30.4
-Multinodular non-toxic goiter (15)	27.7 ± 12.4	24.8	9.9–56.0
-Papillary thyroid carcinoma (3)	NA	NA	NA
TSH (mIU/L)			
-All subjects (100)	3.00 ± 4.29	1.85	0.22–34.50
-Control subjects (25)	1.37 ± 0.81	1.20	0.42–4.20
-Hashimoto’s thyroiditis (45)	3.72 ± 3.00	2.80	0.52–34.50
-Graves’ disease (6)	2.16 ± 1.83	3.50	0.34–4.60
-Nodular non-toxic goiter (6)	1.05 ± 0.61	1.12	0.22–1.70
-Multinodular non-toxic goiter (15)	1.64 ± 1.13	1.60	0.30–4.60
-Papillary thyroid carcinoma (3)	1.80 ± 0.53	1.60	1.40–2.40
Previous steroid treatment for active Graves’ orbitopathy (Y/N)	0/6		
TRAb-positive Graves’ patients (Y/N)	0/6		
Recent exposure to iodinated contrast media (Y/N)	0/100		
Methimazole-treated Graves’ patients (Y/N)	6/0		
LT4-treated Hashimoto’s patients (Y/N)	17/28		

Yr = years, F = female, M = male, Y = yes, N = no, TSH = thyroid stimulating hormone.

## Data Availability

The data presented in this study are available on request from the corresponding authors.
